# LoRaBB: An Algorithm for Parameter Selection in LoRa-Based Communication for the Amazon Rainforest

**DOI:** 10.3390/s25041200

**Published:** 2025-02-16

**Authors:** Diogo Soares Moreira, Gilmara Santos, Angela Emi Yanai, Pedro Barreto de Souza, Paulo Victor Fernandes de Melo, Edjair Mota

**Affiliations:** 1 Institute of Computing (IComp), Federal University of Amazonas, Manaus 69080-900, Brazil; gilmara@icomp.ufam.edu.br (G.S.); pedro.souza@icomp.ufam.edu.br (P.B.d.S.); paulo.fernandes@icomp.ufam.edu.br (P.V.F.d.M.); edjair@icomp.ufam.edu.br (E.M.); 2 Biblioteca Central (BC), Federal University of Amazonas, Manaus 69080-900, Brazil; angela_yanai@ufam.edu.br

**Keywords:** LoRa, LoRa transmission parameter selection, Internet of Things, single-channel LoRa gateways

## Abstract

The interference of human activities in water bodies has contributed to a deterioration in water quality. With the advancement of the Internet of Things (IoT), aided by transmission technologies such as LoRa (Long Range), low-cost solutions have emerged for long-distance environment monitoring scenarios. One key challenge in such IoT-based systems is selecting LoRa transmission parameters to ensure efficient data exchange among nodes, adapting to varying network conditions. Well-known strategies adapt transmission parameters according to network context through information exchange among nodes and LoRa gateway(s). In this work, we introduce a novel LoRa parameter selection algorithm by incorporating three major LoRa metrics (RSSI, SNR, and PDR) and conducting a comprehensive characterization and validation in the forest environment to build a set of reference values of transmission quality, which are employed in a binary search methodology, utilizing the *R*-array, representing the transmission quality according to LoRa parameters. The experimental results indicate that the proposed algorithm achieves a 16.20% reduction in Time on Air (ToA). Furthermore, our algorithm optimized the transmission power (TP) selection, achieving at least 38% lower energy consumption than ADR TP parameters. These results highlight that our proposed algorithm can enhance the transmissions in a rainforest environment.

## 1. Introduction

Water bodies support social and economic activities, such as human consumption, agriculture, industry, and wildlife preservation. However, these resources face increasing restrictions due to pollution caused by natural and human activities. Thus, water usage is subject to significant restrictions for purposes such as human supply [1]. Rapid population growth and urbanization has resulted in elevated pollution levels in regions’ waters [2,3]. Consequently, remote and continuous sensor-based monitoring has become essential.

In traditional water monitoring, samples are collected manually and sent to laboratories for analysis using chemical methods, consuming human, time and financial resources [4]. However, those techniques do not provide real-time insights or low-cost solutions. In contrast, low-cost sensors, integrated into microcontrollers such as Esp32, can be deployed to collect data such as pH, turbidity, temperature, and others, from water bodies in a cost-effective solution for a data collection device [5].

In addressing these challenges, deploying Internet of Things (IoT) technology has emerged as a promising solution for real-time environmental monitoring. Among IoT communication technologies, LoRa (Long Range) and NB-IoT (Narrowband-IoT) have emerged as transmission technologies for IoT systems. While NB-IoT is designed to extend the functionalities of the cellular network to IoT device networks, LoRa uses proprietary modulation to enable low data rates and low-power communication. Although NB-IoT outperforms LoRa in several scenarios—particularly in coverage area due to better utilization of the directional antennas of cellular networks [6]—LoRa offers advantages over NB-IoT in terms of energy consumption during data transmission [7] and reduced development costs for IoT deployments [8]. These factors make LoRa a more suitable transmission option for IoT systems within the environment scope of this project.

Additionally, LoRa offers the possibility of long-distance communication, low power consumption, and cost-effective solutions to the aforementioned IoT scenarios [9]. For these, LoRa utilizes a modulation technique to transmit information using chirp signals on the physical layer [10,11]. Additionally, it provides specific physical parameters to ensure proper communication. These parameters include the folowing:

- Bandwidth (BW): This refers to the communication channel’s capacity to transmit data within a specific time frame. BW significantly influences data transmission speed and sensitivity to noise. For instance, increasing bandwidth enhances data transmission speed but may reduce sensitivity [11].
- Spreading factor (SF): This determines the trade-off between data rate and range in LoRa. Lower SF values provide higher data rates and shorter ranges, suitable for nodes closer to gateways. In contrast, higher SF values are preferable for nodes situated at a greater distance. LoRa defines six SF values (7 to 12) that influence the chirp bit count and directly influence energy consumption and data rate [11,12].
- Coding rate (CR): This determines the bits allocated for error-checking transmission redundancy. In summary, a higher CR value implies a greater degree of redundancy, which contributes to an improvement in the error-correction capabilities of the transmitted data.
- Transmission power (TP): TP represents the transmission power used by the transmitter. Lower TP values contribute to reduced battery consumption, while higher values increase the probability of successful communication and extend the communication range.

Given the potential combinations of these parameters—over 6720 in total [12]—the proper selection of LoRa communication settings is crucial to optimize network performance, minimize packet collisions, and extend the battery life of sensor nodes [12,13,14]. Further, energy consumption can vary—by factors of more than 100 [14]—depending on the selected parameters, highlighting the requirement for careful configuration to ensure the longevity of battery-powered nodes, which could be useful for remote monitoring scenarios.

Deploying LoRa-based sensors in remote regions, including the challenging Amazon region, offers a cost-effective alternative, despite the geographical and environmental challenges, including non-line-of-sight (LoS) communication and dense forest cover [15].

**Figure 1:**
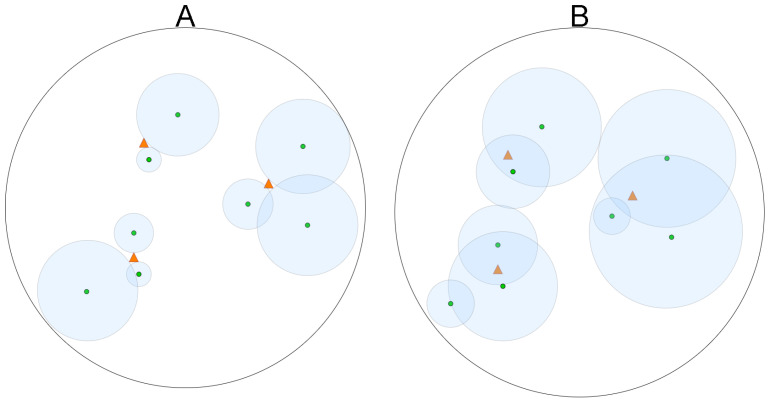
LoRa parameter selection: (**A**) illustrates optimal parameters, while (**B**) depicts suboptimal choices. Each smaller circle represents a node, while the larger circle denotes the communication range determined by the selected LoRa parameters. Triangles represent gateways. Communication can occur between nodes and gateways that are within or on the border of the larger circle.

These geographical features and communication through forests contribute to distinct interference levels. For instance, Figure 1A illustrates an example of LoRa communication among devices when the LoRa parameters are set to the optimal values for communication with gateways, represented by red triangles. At the same time, in Figure 1B, incorrect parameter selection can lead to suboptimal configurations and waste of energy resources [12,14].

Despite the recent literature about the usage of LoRa in IoT scenarios [16], we have found a lack of research studies on LoRa parameter selection, such as the absence of combining multiple LoRa parameters and the need for experimentation in real and challenging environments such as remote areas or forest areas [17]. On the other hand, Ballerine et al. [8] describe that LoRa parameter selection strategies such as ADR are relevant for mitigating deployment costs and energy consumption in comparison to NB-IoT systems. These research gaps highlight the requirement for researchers to consider non-line-of-sight (LoS) conditions typically found in forest environments.

In response to these challenges, this paper offers the following major contributions: (a) it proposes a novel algorithm as one of the first works in the literature to incorporate the three major LoRa metrics (received signal strength indicator (RSSI), signal-to-noise ratio (SNR), and packet delivery ratio (PDR)) in the parameter selection algorithm design; (b) it presents an *in situ* rainforest transmission characterization and validation conducted in a real and challenging Amazon rainforest scenario; and (c) it proposes an algorithm for selecting LoRa parameters using the traditional binary search methodology. This approach provides an accurate representation of LoRa transmission in a challenging scenario such as that experienced in the Amazon region.

The proposed algorithm, entitled LoRaBB (Lora Parameter Selection via Binary Search), utilizes an *R*-array of reference values from key LoRa metrics—RSSI, SNR, and PDR—by combining the following LoRa parameters: SF, BW, and PT. The *R*-array, embedded in the source code of each deployed node, serves as a benchmark. During the LoRa parameter selection phase, each node compares its current communication metrics with these reference values to identify feasible arrangements for the parameters selected. Finally, the proposed algorithm compares parameters and metrics, employing a binary search methodology, fully described in Section 3.1, until they are appropriately adjusted to optimal values.

To validate and test the LoRaBB algorithm, we conducted characterization experiments to evaluate LoRa transmission in a forest scenario, with low- or non-line-of-sight (LoS) conditions. The results were evaluated to verify if the strategy of a *built-in R*-array is suitable for representing, in a numerical form, the combination of selected parameters in a LoRa transmission between nodes.

The results demonstrate that the LoRaBB algorithm can fit into a real-world forest communication scenario, encompassing different distances and levels of interference based on the LoRa parameter combination. In addition, the results show that LoRa is more suitable for forest scenarios when compared to the ADR algorithm used in LoRaWAN, achieving a Time on Air (ToA) 16.20% lower, and lower theoretical energy consumption during transmission.

The rest of this paper is organized as follows: Section 2 presents the related work found in the literature, while Section 3 presents a general overview of the architecture that supports the solution proposed, as well as the proposed algorithm. Section 4 presents the design of the experiment to validate the proposed algorithm. Finally, Section 5 and Section 6 present the major results and discussion, as well as the conclusions and future work.

## 2. Related Work

Previous studies on LoRa have focused predominantly on the applicability of LoRa in IoT scenarios and applications, particularly in contexts such as smart cities, animal tracking, environment monitoring, and other scenarios [18,19].

In LoRa transmissions, several parameters must be adjusted to ensure the link transmission quality between nodes or between nodes and gateways, optimize energy consumption, and improve network performance. Thus, these parameters must be used for (i) link estimation quality: when the nodes estimate the communication quality based on LoRa metrics such as RSSI, SNR, and PDR; and (ii) parameter selection: when nodes adjust transmission parameters based on the assessed communication quality [20].

LoRa devices can be configured with different values of SF, BW, CR, and TP from 0 dBm to 14 dBm (or 20 dBm in some devices) [16]. Li et al. describe that parameter selection can be influenced by environmental factors, necessitating consideration of trade-offs between packet delivery ratio and energy consumption [16]. These insights align with other research findings, indicating the need to adapt transmission parameters according to deployment scenarios [16,21,22,23].

For instance, Gao et al. [24] demonstrate that the distance between the transmitter and receiver should lead LoRa parameter selection to achieve a low data rate and increase the chances of delivery, particularly over greater distances. Moreover, Angrisani et al. [25] evaluated that, although LoRa is highly robust to high noise levels, the variations in the parameters can impact packet losses over different values of SF, CR, and BW. Cattani et al. further concluded that the packet delivery ratio and the parameter selected are on a narrow boundary, where the packet delivery ratio was only 10% lower than that of the slowest LoRa parameter selected when a node was distanced from the gateway [23].

Another notable LoRa parameter selection strategy is the Adaptive Data Rate (ADR) strategy, initially implemented by The Things Network (TTN) according to Semtech’s development recommendations [26]. The ADR adjusts LoRa parameters based on the SNR values from the most recent packets received during the algorithm’s execution. To address it, the gateway listens for frames transmitted in multiple frequency channels. However, as highlighted by Wang et al. [20], the ADR adds computational complexity to the gateway’s processes. In a low-cost system, as discussed in this paper, increased computational complexity can lead to a costly gateway build process.

To automate the selection of LoRa parameters, Serati et al. [27] have developed an algorithm, entitled ADR-Lite, which employs a binary search methodology. The ADR-Lite considers four parameters, SF, TP, carrier frequency (CF), and CR, using the packet delivery ratio as the query key, to identify the most suitable setting to achieve improved PDR values. Moreover, an upper bound for energy consumption values is considered, specifying that the parameter chosen must adhere to this criterion to be considered as the optimal choice. However, the strategy design did not consider RSSI and SNR factors in the algorithm design, and even the BW parameter, which plays an important role in energy consumption, was not incorporated into its combination of parameters.

Following a similar approach, de Jesus et al. proposed the algorithm ADR-X [28] by slightly modifying the classic ADR, incorporating an adaptive threshold representing the acceptable SNR level to compensate inaccurate estimation of the transmission quality, such as found in lossy channels. Unlike the traditional ADR, this threshold is automatically adjusted for each node.

The research by Khalifeh, Shefaa, and Darabkh [29] presented an algorithm that enables the dynamic exchange of channels to select the option with the least traffic and choose the most appropriate SF to support the data rate demand in LoRa communications. Moreover, the aforementioned research presented a need for experimentation in real environments; it is a centralized solution, not suitable for some IoT scenarios. Further, the details of the algorithm design were not fully given.

Moreover, a recent trend, widely disseminated in the literature, involves the application of machine learning (ML) techniques to select smart LoRa parameters. In the research referenced [30,31], the authors investigated this methodology that prioritizes energy efficiency and improves the PDR using conventional ML approaches. Similarly, Ilahi et al. [32] applied a deep learning algorithm formulated as a Markov decision process to optimize the PDR in the ADR process.

Finally, as evidenced in the related works analyzed, the current research focuses on performance evaluation within simulated environments. In addition, there is a gap concerning the study of parameter selection in wooded contexts, particularly in scenarios such as Amazonian rainforest regions. Table 1 presents a summary of the main literature research analyzed, the LoRa parameters used in their research, and their scenario conditions.

## 3. Architecture Overview

The architecture and all the protocols and algorithms presented in this work were developed using ESP32 Heltec V2 devices (as End-node devices) and V3 devices (as gateways). Each one of these devices was equipped with SX1276 and SX1262 modules, respectively. To enhance signal quality, Steelbras AP3900 antennas with 5 dBi gain were utilized. Furthermore, the data generated during samplings were stored using a Raspberry Pi 4 Model B and a Pi 3 Model B+ in gateway and end-node devices, respectively. To clarify, in the context of this paper, each end-node device is integrated into a water sensor and transmits a C-style structure payload of 20 bytes over LoRa. Considering that only packets with information about data collected are sent, there is no reasonable reason to explore higher payloads sizes.

Further, it is important to highlight that LoRaWAN capabilities such as LoRaWAN gateways were not deployed due to the unavailability of LoRaWAN gateways in the Amazonas region. Further, using microcontrollers and shields to deploy LoRaWAN gateways could have increased the costs of the project contextualized in this paper up to 10 times.

The following subsections detail the establishment of LoRa parameters between end nodes and gateways, providing an overview of ADR algorithm, and describing the operational process of the proposed LoRaBB algorithm.

### 3.1. Overview of Technique Requirements

The communication protocol is designed based on the requirements that both LoRaBB and ADR must fulfill. In addition, this research focuses on two components of a LoRa network, as described by LoRa Alliance [33]: End devices and LoRa gateways. The following assumptions guided the system architecture and the experiments.

- Stationary end nodes: The end nodes, also known as end devices, contain a LoRa transceiver and transmit data to gateways using LoRa modulation [33]. Each node deployed remains stationary, serving as a communication network endpoint. However, we have considered that its initial location is initially unknown to both nodes.
- Node-gateway discovery: Gateways are nodes with Internet connectivity, consisting of a transmitter and a microprocessor [33]. Further, the gateway is designed to attempt to initiate the LoRa parameter selection by using a set of default parameters, facilitating the node discovery phase.

We assume that algorithms can be triggered to initiate when gateways and end nodes have the same set of default parameters (DPs), enabling a three-way handshake.

**Figure 2:**
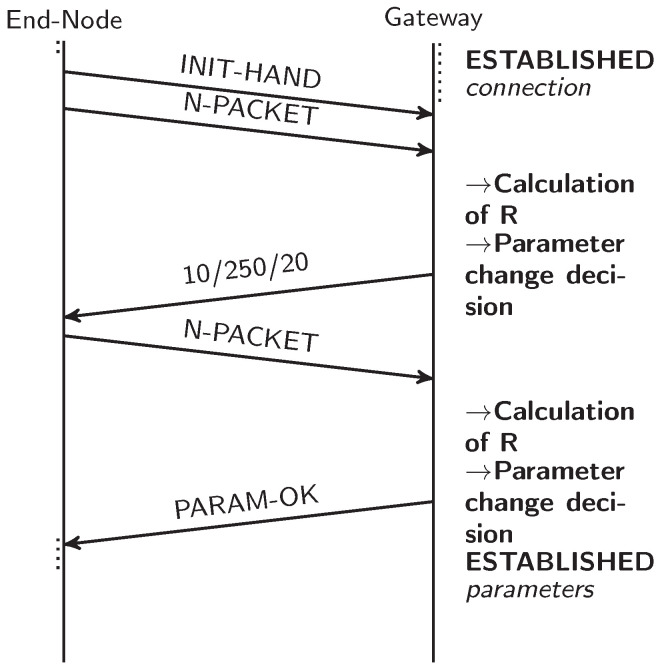
Three-way handshake to establish first contact between an end node and a gateway.

A three-way handshake was employed to establish the device connection and initiate the algorithm steps. To establish the initial exchange of messages, the DPs were adopted, as summarized in Table 2, as they theoretically provide a more robust setting for data transmission. This handshake mechanism allows devices to verify compatibility and ensures the synchronized adjustment of their parameters during communication. Figure 2 presents this whole process, concisely describing the preliminary algorithmic steps.

Moreover, each gateway is designed to retain information about LoRa parameters to facilitate the communication with other nodes, periodically adjusting its settings for data transmission and new node discovery.

### 3.2. ADR LoRaWAN

The Adaptive Data Rate (ADR) algorithm from The Things Stack is a widely known strategy for optimizing communication in LoRaWAN networks. The ADR analyzes the SNR to determine the most appropriate data rate and transmission power settings for the network devices [34].

**Figure 3:**
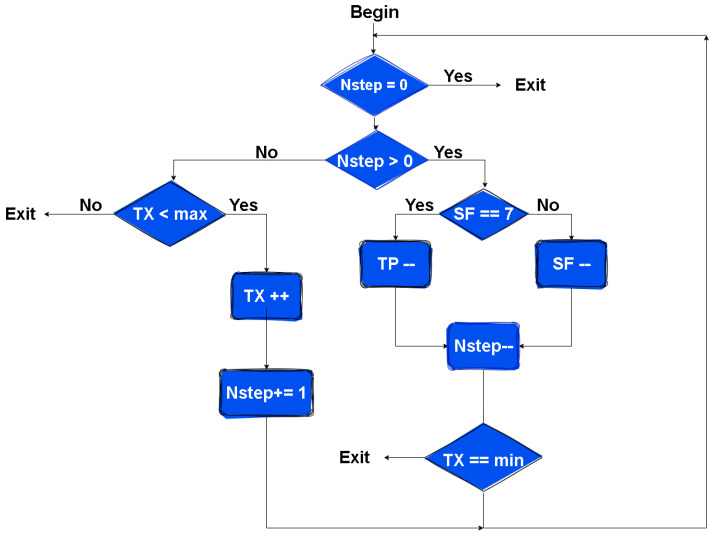
ADR algorithm flowchart [34].

Equation (1), in the first phase of ADR execution, determines the number of steps performed in the algorithm, as illustrated in Figure 3. Thus, the gateway estimates the maximum SNR value (${\mathrm{SNR}}_{\max}$) of the last *N* transmissions, while the value of ${\mathrm{Margin}}_{\mathrm{db}}$ varies between 5 and $15_{\mathrm{db}}$ [34]. In our implementation of the algorithm, we set this to $15_{\mathrm{db}}$. The ${\mathrm{SNR}}_{\mathrm{sf}}$ represents the SNR required for successful demodulation.

(1) $$N_{\mathrm{step}}=\frac{{\mathrm{SNR}}_{\max}-{\mathrm{SNR}}_{\mathrm{SF}}-{\mathrm{margin}}_{\mathrm{dB}}}{2.5}$$

### 3.3. The Proposed LoRaBB Parameter Selection Algorithm

Optimizing the trade-off between convergence time toward the optimal configuration and parameter selection drove the decision to employ the binary search methodology in our proposed algorithm. Therefore, we designed the proposed algorithm in two main phases:

1. R-array building: In this phase the communication in the forest scenario is characterized and evaluated in order to create the *R*-array, a structure that defines the mean expected performance of the LoRa communication in our scenario. The main aspects of the scenario characterization and the details of the *R*-array building are described in Section 3.3.1.
2. Algorithm design: In this phase, the algorithm is fully described, as well as how each end node uses the *R*-array loaded in its firmware to run the algorithm logic during the life cycle of the network. The details of how the binary search approach was utilized is described in Section 3.3.2.

#### 3.3.1. The *R*-Array Building

Initially, we built an *R*-array using data collected from practical experiments conducted in a forest environment. This array is an instrument to evaluate the relationships between RSSI, SNR, and PDR relating to SF, BW, and PT values in our forest scenario. In summary, it is a method of representing the expected communication performance over different parameter combinations in a scenario. Moreover, we followed some consolidated LoRa theories to build the *R*-array: (a) higher SF produces higher reliability, resulting in lower receiver sensitivity and increasing the probability of packet delivery [35,36]; and (b) Time on Air doubles the transmission duration at each increase in SF [36]. Therefore, LoRaBB represents these affirmations in a single value for each parameter combination, called *R*.

Each *R* entry in the *R*-array represents a value derived from a reward equation called *R* (Equation (2)). Assigning weights (W1, W2, W3) to each metric, so, RSSI, SNR, and PDR exhibit distinct patterns for specific parameter combinations. Additionally, RSSI, SNR, and PDR values used in both the *R*-array and algorithm design undergo normalization, ensuring a standardized range of 0 to 1, using the formula $\frac{V_{value}-V_{max}}{V_{min}-V_{max}}$, such that V= {RSSI, SNR, PDR}. Finally, it is worth highlighting that the computation of the *R* value for each parameter combination during this phase is performed using the average of the RSSI, SNR, and PDR values found in the experiments. In the equation, this is represented by the overbar symbol.

(2) $$R=W_{1}\cdot \bar{RSSI}+W_{2}\cdot \left(\bar{{\mathrm{SNR}}_{i}+20}\right)+W_{3}\cdot \bar{PDR}$$

To build the *R*-array, two ESP32s were placed within the campus of the Federal University of Amazonas (UFAM) to generate the results of data communication within the forest. As stated in Section 1, the UFAM campus is one of the largest urban forest environments, which is suitable for any characterization and further validation in this project’s context. The data collected characterized the Amazon rainforest environment and established the base *R*-array used as a parameter for LoRaBB.

The ESP32 modules, designed as transmitter and receiver (similarly to the end node and gateway behaviors, respectively), were placed at different locations and separated by significant distances to exchange data through an area of vegetation typical of the Amazon rainforest. These characterization experiments occurred in sunny conditions; the detailed parameter values are provided in Table 3.

**Figure 4:**
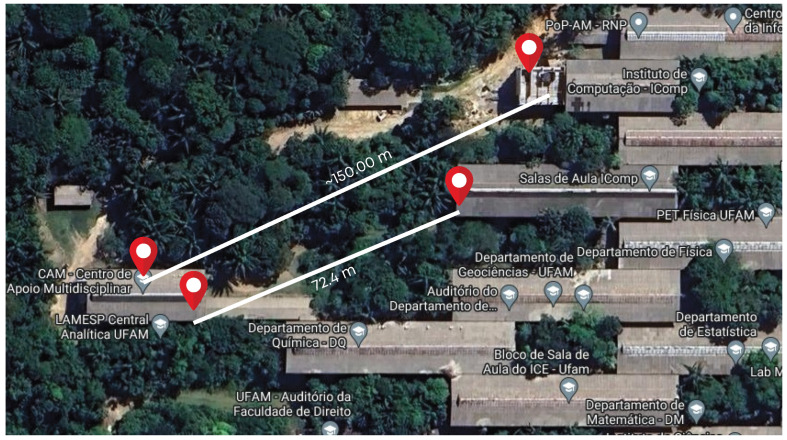
Location of experiments for *R*-array building.

In the initial experiment, the transmitter was situated at a height of approximately 12 m, on the third floor of the Institute of Computing (IComp) building (*Salas de Aula Icomp* on the map), while the receiver was positioned at a height of approximately 8 m, on the second floor of the Analytical Chemistry building. Twenty packets were transmitted for each parameter combination, covering approx. 72 m during this stage. Subsequently, the transmitter device was relocated to the IComp2 (*Instituto de Computação—IComp* on the map) building for a subsequent experiment, extending the distance to approx. 150 m. The experimental placements are illustrated in Figure 4 (the full dataset of the characterization experiments is available at https://doi.org/10.48472/deposita/FRRQ8R, accessed on 8 January 2025).

**Figure 5:**
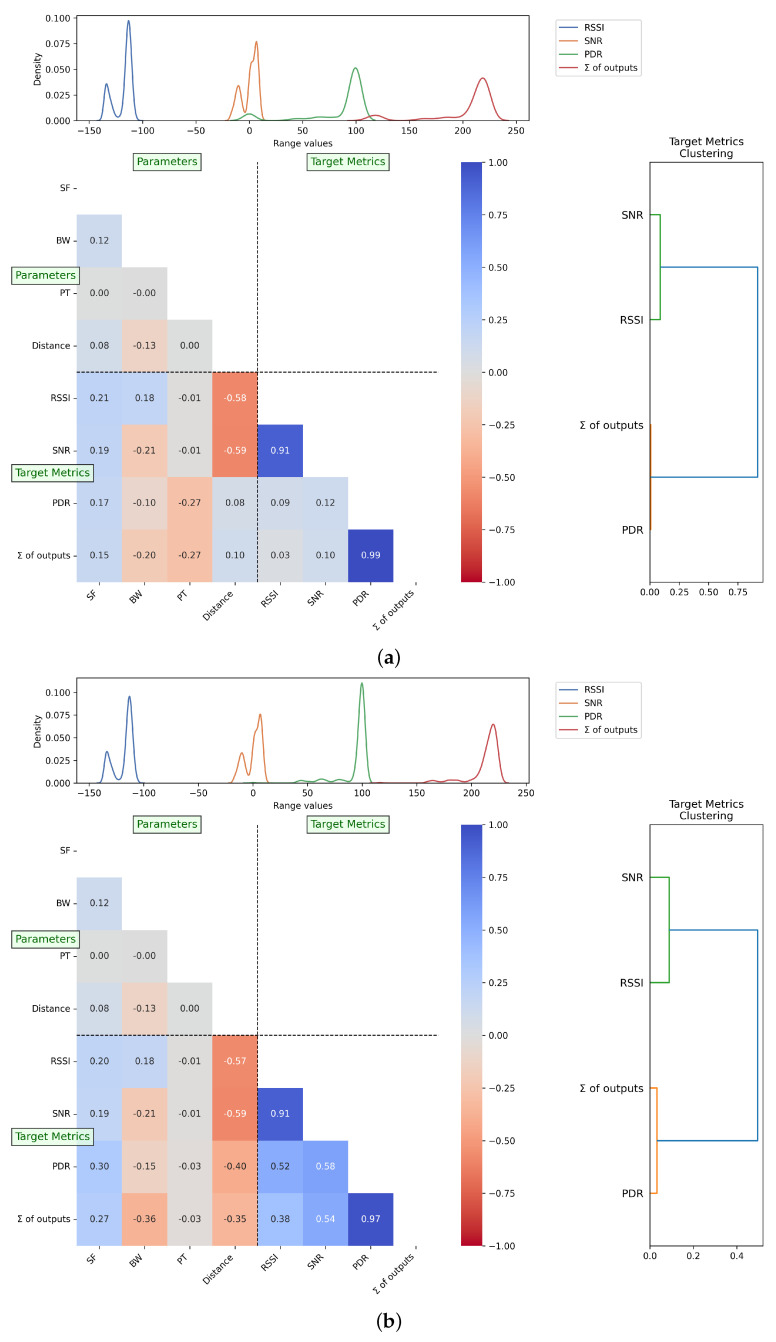
Correlation matrices illustrating the relationship between parameters and target metrics in the R-array characterization process. (**a**) Correlation matrix including the PT = 18 scenario; (**b**) correlation matrix excluding the PT = 18 scenario.

The results from practical experiments illustrate that the behavior of the evaluated metrics (RSSI, SNR, and PDR) can be influenced by the values of SF, BW, and PT, as shown in Figure 5a. In addition, another value was added, named the ∑ of the outputs, a straightforward summation of the normalized RSSI, SNR, and PDR values. This underscores the appropriateness of our *R* characterization methodology in modeling the parameter selection algorithm.

At this point, we highlight specific aspects of the characterization, particularly the case where PT = 18. For some anomalous reason, most cases where PDR was below 50% or equal to 0% occurred when PT = 18 for both characterized distances. At first, we hypothesized that this might be due to experimental errors in the first experiment, but we also found five cases where PDR < 50% and PT = 18 in the second experiment (longer distance). In the first characterization experiment, we found 17 cases where the LoRa parameter combination with PT = 18 had PDR = 0%.

As shown in Figure 5a, this result challenged common sense and contradicted the theoretical expectations of LoRa communication assumptions between the following target metrics and parameters: the positive correlation between PDR and distance, and the negative correlation between PDR and PT. To address this anomaly, we investigate the dataset excluding the parameter combinations where PT = 18.

As illustrated in Figure 5b, without this scenario, the correlation between PDR and PT, as well as PDR and distance, aligns with LoRa theory. Specifically, we observe a negative correlation of −0.40 between PDR and distance, alongside a reduction in the negative correlation between PDR and PT, with a negligible correlation showing that varying PT in our scenarios does not impact the results, unlike when varying SF or BW. This is probably due to the fact the overall mean PDR was approximately 85% across all PT values, except for PT = 18, where the PDR dropped significantly to around 40%.

Since this behavior was observed across both distance scenarios where PT = 18, we consider two possible hypotheses: the influence of small-scale fading caused by multipath phenomena and near-point obstacles [37], or a compatibility issue between the ESP32 modules and the C library used in the experiments.

On the other hand, SF and BW are aligned with the expected performance observed in other LoRa studies. Applying a one-way ANOVA test, we confirmed that the null hypothesis can be rejected for the means observed, with a *p*-value = $1.560^{-219}$ for the SF–PDR relationship and *p*-value = $3.994^{-74}$ for the BW–PDR relationship.

**Figure 6:**
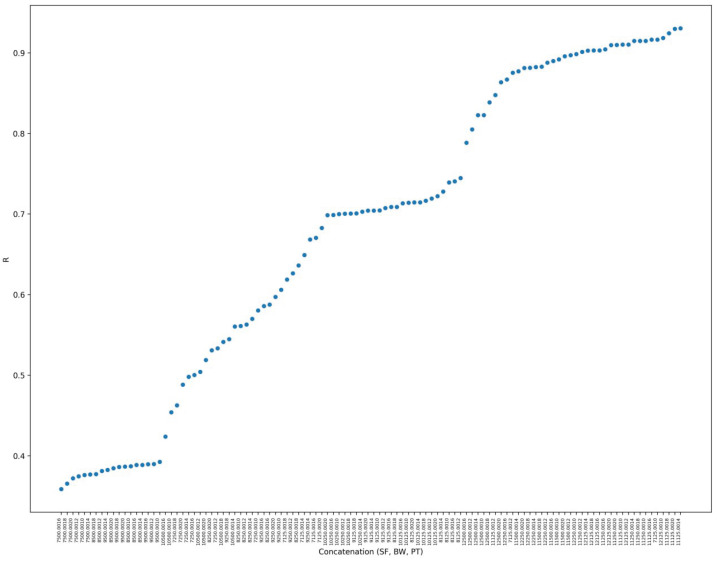
R values found in characterization experiments for each parameter combination. A very high resolution version of the R chart is available at: https://tede.ufam.edu.br/image/R_values_characterization.jpg, accessed on 15 December 2024).

While factors such as distance negatively affect RSSI and SNR, these metrics can be directly influenced by the choices of SF and BW. To better visualize this, Figure 6 presents the impact of parameter selection on the *R* value, where each parameter combination on the x-axis is represented by a concatenated string of the SF value, BW value (e.g., 125.00 MHz), and PT. The corresponding *R* values are plotted on the y-axis.

Moreover, since PT was identified to have a low correlation with RSSI, SNR, and PDR, the chosen PT values for each parameter combination are spread over Figure 6. Furthermore, to clarify, the left values on the plot where R≤0.4 result in close to 100% packet losses and they represent the worst expected scenarios. These results corroborate our hypothesis that ADR is not the most suitable strategy for LoRa parameter selection since transmission power is one of the parameters used in the strategy.

Figure 6 also demonstrates that parameter choices yield different *R* values, which can have implications, such as in energy consumption or PDR. For instance, suppose a parameter combination that generates an *R* value close to 1 is chosen for a specific communication. When analyzed, it becomes evident that this parameter selection tends to favor values that could maximize delivery possibilities, especially with higher SF and lower BW values, which directly impact PDR and energy consumption, for example.

Subsequently, the *R*-array is sorted based on the value of *R* entry and deployed into the firmware code of each deployed end node. It is important to highlight that the first step of this work demonstrated that the values in the *R*-array could not follow the usual distribution of a LoRa parameter combination. This irregular pattern may be attributed to signal attenuation and environmental factors such as dense foliage, which can affect certain SF values, especially when SF is between 8 and 11, as stated in the aforementioned *R* -value chart, where it can be observed that SF combinations are spread over the middle part of the *R*-value chart.

**Figure 7:**
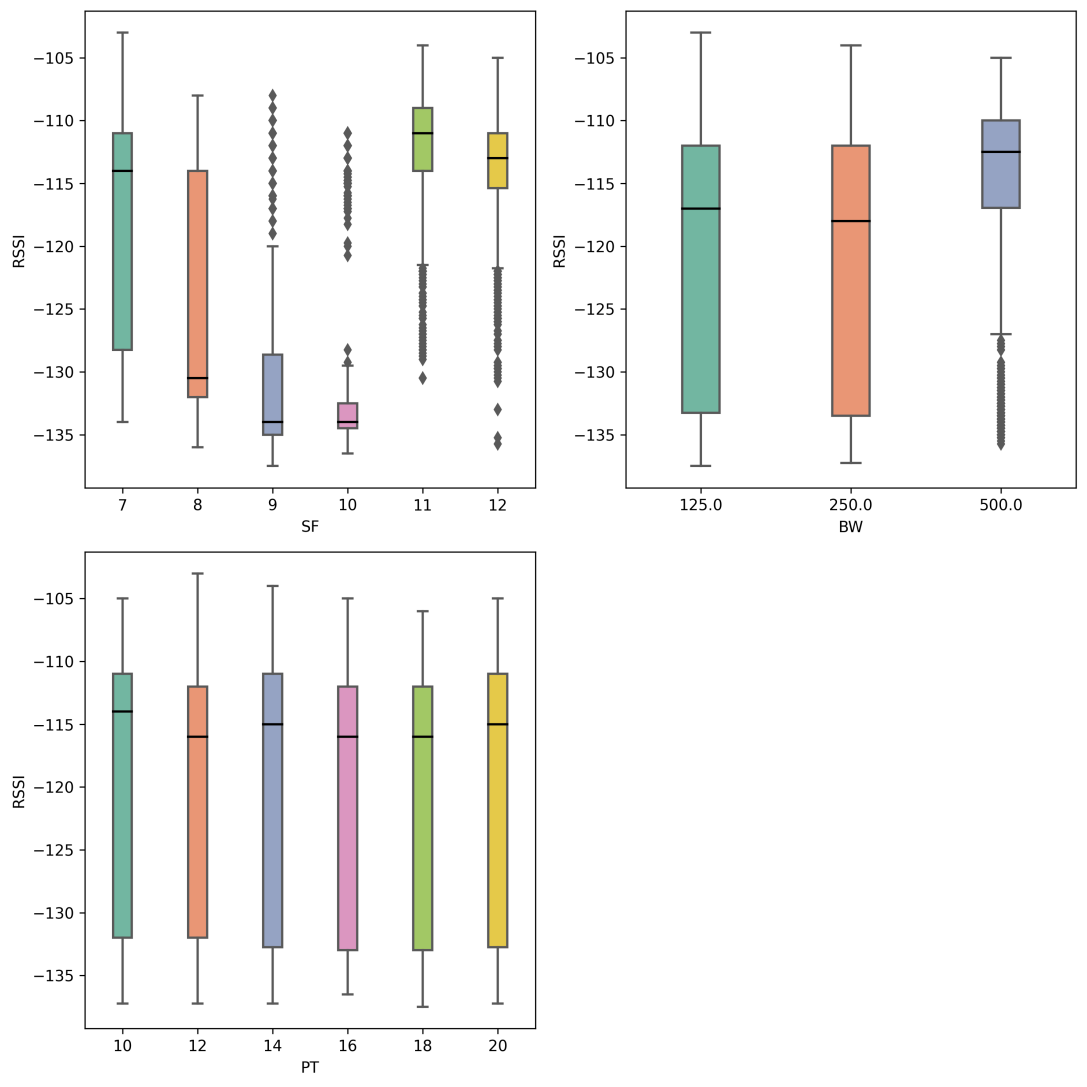
RSSI results in the *R*-array build characterization experiment for each LoRa parameter.

Although this behavior may seem unusual, it has been observed in previous studies with forest environments, e.g., by Ansah et al. [36], where the authors found higher SF (SF = 11 and 12) values produced less ToA and extended range than lower SF values from 7 to 9. Our findings corroborate this, as demonstrated in Figure 7, which presents the average RSSI values found for each parameter in all characterization runs. The characterization results demonstrate SF values of 9 and 10 produced the best sensitivity values in the forest environment, while transmission power had a low impact at the distances tested in the experiments.

Furthermore, our results align with the findings of Ferreira et al. [37]. The authors could not explain the behavior of PDR and its relationship with RSSI and SNR depending on the SF parameter in forest environments. They attributed this fact to small-scale fading due to multipath phenomena and near-point obstacles.

**Figure 8:**
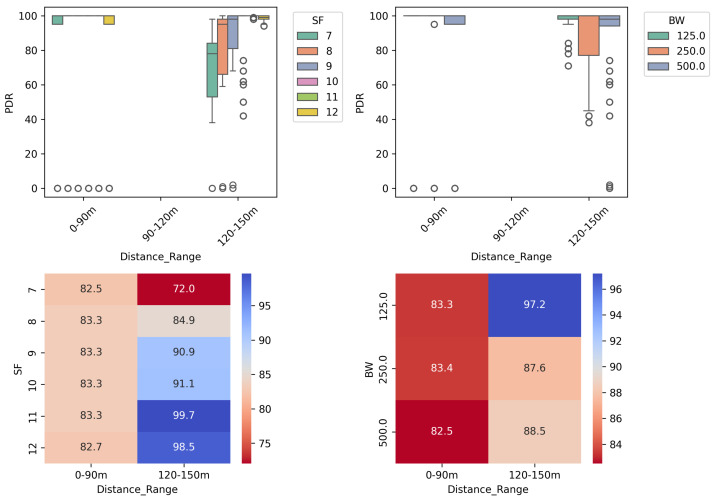
PDR results in the R–array build characterization experiment for each combination of SF and BW parameters at varying distances.

This anomalous behavior was also observed in the correlation between **distance** and **PDR**, in relation to SF and BW values. As shown in Figure 8, the PDR was unexpectedly lower in almost all cases at a distance of 72 m. These results further support the positive correlation found between **distance** and **PDR**. Additionally, it was observed that deviations in PDR values increase at longer distances. This result is consistent with the RSSI deviation described in Figure 7, where the RSSI variation led to outliers found in the PDR results.

#### 3.3.2. The Binary Search Phase

The algorithm execution comprises three primary steps: the initial step, the *R* evaluation step, and the optimal LoRa parameter selection step.

In the initial step, the algorithm initiates its process with the DP configuration, as detailed in Section 3.1. Notably, communication is established before testing the parameter selection. Further, it is worth highlighting that gateways overhear the DP configuration, initiating the LoRa parameter selection binary search algorithm for each newly deployed node.

During the *R* evaluation step, the algorithm transmits *N* packets from the node to the gateway. It then employs Equation (2) to compare the calculated R to the *R*-array. The comparative analysis determines whether the calculated *R* for the current parameter combination aligns with empirical results or if parameter modifications are necessary to test and identify a more suitable combination. When adjustments are needed, the configuration is adjusted to the left or right side of the *R*-array, using the classic binary search methodology, iterating through packet transmission and assessment until convergence on the appropriate configuration is achieved.

**Figure 9:**
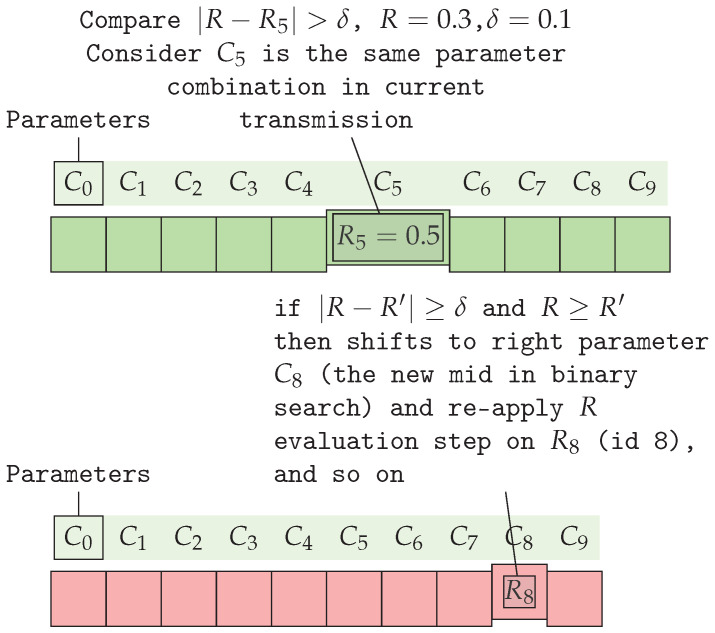
Example of binary search algorithm proposed using *R* values in sorted *R*-array for the calculated R=0.3, by comparing them in the green *R*-array using δ=0.1. In the red *R*-array, the old parameter combination ($C_{5}$) has shifted to the right side of the array to use the new parameter combination ($C_{8})$, and comparing it to the newly calculated *R*, re-applying the *R* evaluation step using $R_{8}$.

Figure 9 illustrates an instance of the algorithm’s R evaluation step. To simplify, assume the parameter combination $C_{5}$ is tested first (in the LoRaBB algorithm, the first combination is typically represented by the DP configuration).

The *R* value for $C_{5}$ follows the *R* evaluation step by using the LoRa parameters SF, BW, and PT. This value, denoted as $R^{\prime }=R_{5}=0.5$, is compared to R=0.3, the current calculated *R*. If the absolute difference $R-R^{\prime }$, where $R^{\prime }\in R$-array and $R^{\prime }=R_{5}=0.5$, exceeds the threshold δ=0.1, the algorithm determines the next step:

1. Initially, when $R<R^{\prime }$: In this condition, the algorithm detected suboptimal network performance, since $R<R^{\prime }$. Thus, LoRaBB indicates that it might be a more suitable LoRa parameter combination for the end node, by improving LoRa parameters such as increasing SF or PT, or decreasing BW. Consequently, it shifts to the right side of the *R*-array using the binary strategy.
2. Next evaluation: Following the shift step, the LoRaBB calculates the new *R* value for the combination represented by $C_{8}$ in the *R*-array, yielding R=0.95.
  Comparing it to the updated $R^{\prime }=R_{8}=0.8$, if the absolute difference is lower than the δ, the algorithm will shift to the left side to search for a more suitable parameter combination since *R* is greater than $R^{\prime }$, so the parameters can still be optimized.
3. Stopping conditions: The search phase concludes when either the absolute difference between *R* and $R^{\prime }$ is lower than δ or no further binary steps are available.

To clarify, this example can be expanded to include more details about how LoRaBB works by considering the following assumptions: (a) $C_{5}$ is the first combination parameter to be tested and it represents the following parameters in this example: SF = 8, BW = 500.00, and PT = 20; (b) consider the distances between nodes are short enough to choose an energy-efficient parameter configuration; (c) these parameters yield an initial calculated R=0.3, due to low PDR; (d) based on the $|R-R^{\prime }|\geq \delta$ condition, it tries to estimate if the binary search will move to the left, to the right, or if the parameter will be accepted. Therefore, consider the three steps of execution for this instance, presented in Table 4.

Considering the aforementioned step-by-step example shown in Table 4, the process begins with parameter combination $C_{5}$, using SF = 8 and BW = 500 kHz. During operation, these parameters yield a calculated *R* value of 0.30, which differs significantly from the expected *R*-array value of 0.50. Since |0.30−0.50|=0.20 exceeds the threshold δ=0.1, the algorithm moves to the right in the *R*-array to test the new middle $C_{8}$.

Therefore, the second step evaluates $C_{8}$ with SF = 12 and BW = 125 kHz, resulting in a calculated *R* of 0.95, compared to the *R*-array value of 0.80. While this indicates better performance than expected, the difference still exceeds δ, prompting the algorithm to move left to $C_{6}$ to look for a more energy-efficient parameter combination. The final test, with SF = 10 and BW = 500 kHz, yields a calculated *R* of 0.75, closely matching the *R*-array value of 0.70. Since |0.75−0.70|=0.05≤δ, these parameters are accepted as suboptimal for the current deployment conditions.

**Figure 10:**
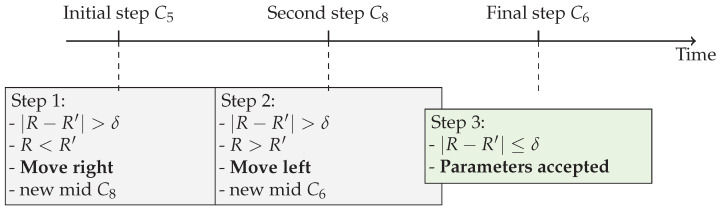
Timeline visualization of the LoRaBB binary search phase process, showing the progression and decisions at each step.

This example demonstrates how LoRaBB adjusts parameters until finding a combination where the actual performance aligns with the characterized expectations, rather than simply maximizing the *R* value, which could set a non-energy-efficient parameter combination. The timeline visualization in Figure 10 provides an additional perspective on the temporal progression of the parameter selection process.

At this point, it is highlighted the primary objective of the LoRaBB is to select the parameter combination that most aligns with the average performance across the scenario characterization of the end-node deployment based on the evaluation of the *R* value. The selection of the most appropriate parameters is determined by comparing *R* values, which can influence the network performance. It is highlighted that the weights $W_{k}$ can impact both the characterization and the evaluation of LoRaBB. Since PDR is the most critical metric for the context of this project, PDR was assigned a greater weight value of 60%. These values were determined after characterization results involving the building of the *R*-array.

**Figure 11:**
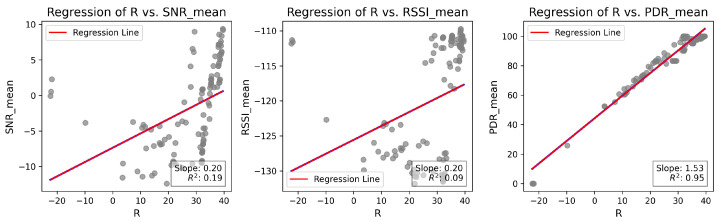
Linear regression analysis of *R* values compared to the means of SNR, RSSI, and PDR for each combination/distance during the characterization experiments, considering $W_{k}=0.33$.

Figure 11 compares the *R* when weights $W_{k}=0.33$ were applied in *R*-array construction, as described in Equation (2). These results were extracted from network characterization experiments and were analyzed to define the most appropriate weight values. As observed, PDR presents the strongest correlation with *R* when the weights are equal. The primary objective of LoRaBB is to select the combinations that align with the average performance presented in the characterization. Further, PDR is the primary metric requirement considered for this project, so these weights were tested prior to choosing the values used in the design of experiments.

**Figure 12:**
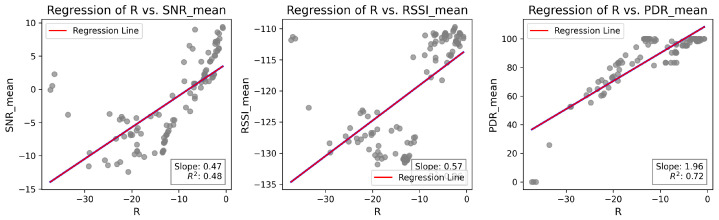
Linear regression analysis of *R* values compared to the means of SNR, RSSI, and PDR for each combination/distance during the characterization experiments, considering $W_{1}=W_{2}=0.2$ and $W_{3}=0.6$.

Following the analysis of *R* values, the weights $W_{1}=W_{2}=0.2$ and $W_{3}=0.6$ were considered for algorithm design and design of experiments, as they provide the most appropriate condition to estimate *R* in the characterized scenario. This condition is illustrated in Figure 12. Similar to the case with equal weights, the slope and the regression of *R*, compared to PDR, are higher, and the coefficient of determination R demonstrates a stronger correlation with *R*. In practice, when nodes choose different parameter combinations, the LoRaBB strategy in this phase will try to overfit the parameter selection process based on *R* values, especially when using PDR as the main metric.

Finally, the complete and detailed algorithm of the binary search proposed can be seen in Algorithm 1.

**Algorithm 1:** Binary Search LoRa Parameter Selection.

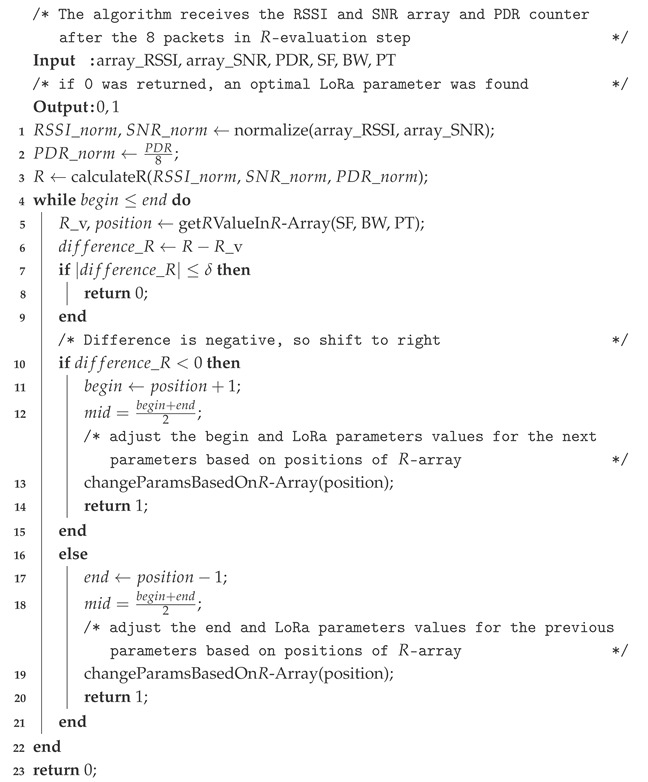



## 4. Design of Experiments

**Figure 13:**
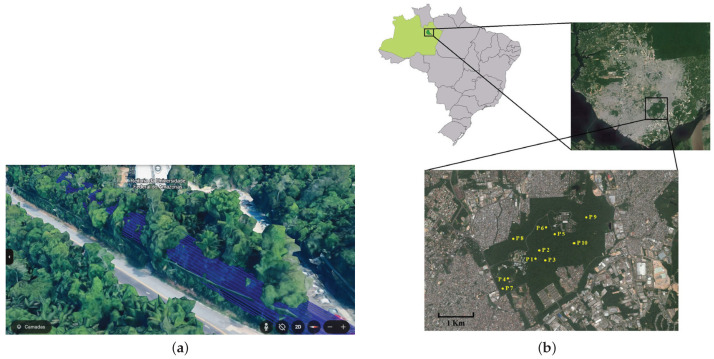
Illustrations about the scenarios used in this research on the campus of UFAM. (**a**) The view of line-of-sight transmission across the forest at UFAM campus. (**b**) UFAM campus, one of the world largest urban forests, in the middle of Manaus City [38].

In the context of this project, it is relevant to highlight that the communication must include transmission across forested environments. Figure 13a illustrates an example of the line-of-sight communication used in this work’s experiments. The image was taken on the campus of the Federal University of Amazonas (UFAM), located in Manaus, Amazonas, Brazil (03º04′34′′ S, 59º57′30′′ W; datum = WGS84). Despite being situated on a university campus, this environment likewise illustrates the same conditions of real-world experiments in the Amazon rainforest. UFAM hosts one of the largest urban forest fragments of the planet, a 776 ha campus mainly composed of primary and secondary forest, as well as anthropic areas and water bodies, as illustrated in Figure 13b, taken from [38].

**Figure 14:**
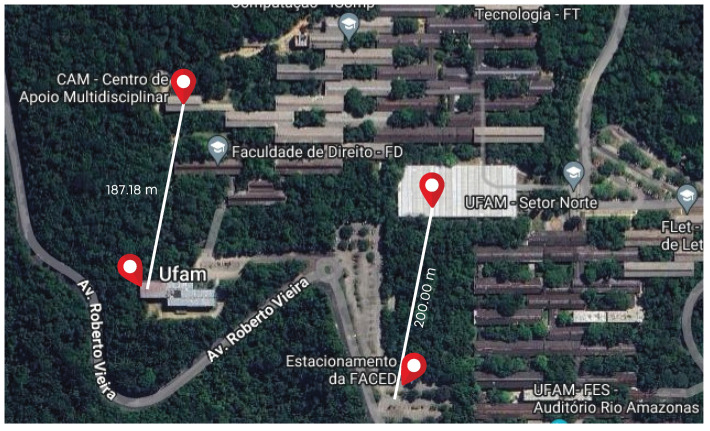
Location of validation experiments for proposed algorithm effectiveness.

To test the effectiveness of our proposed algorithm, we experimented to compare its performance with the traditional ADR approach. The experiments were conducted in vegetation and climate conditions similar to the experiments used to build the *R*-array, described in Section 3.3.1, except the distances of 187 and 200 m, respectively. Moreover, each experiment consisted of the initial stage described in Section 3.1. The decision to use similar scenarios was motivated by the familiarity with the potential outcomes of the characterization experiments. This familiarity facilitated the subjective qualitative analysis concerning the suitability and comprehensibility of the potential results employed in the phase of *R*-array building. The detailed location of the validation experiments is illustrated in Figure 14.

The metrics evaluated were (a) convergence time, representing the duration for the algorithms to select optimal parameters; (b) packet delivery ratio, representing the number of packets delivered in a communication after LoRa parameters was selected by the proposed algorithm and ADR; and (c) qualitative analysis of the chosen parameters.

Weight values were assigned based on the results obtained from the characterization experiments. The weights used for the calculation of *R* were 0.2 for RSSI and SNR and 0.6 for PDR in Equation (2). The weight choices aim to substantially elevate the significance of PDR by choosing a parameter combination focused on minimizing packet loss.

## 5. Results

The results evaluated LoRaBB compared to ADR LoRaWAN for the aforementioned devices set up in forest transmission conditions. In the following charts, the numbers following the names ADR and LoRaBB represent the location of the experiment, as described in the previous section.

**Figure 15:**
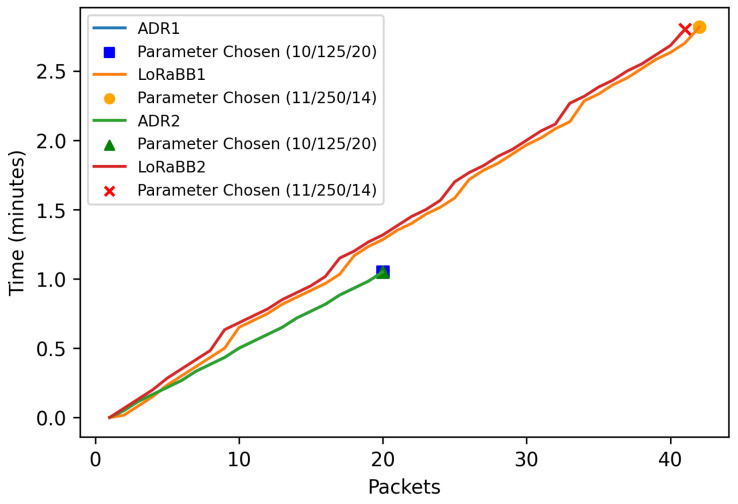
Convergence time for select LoRa parameters.

The results for convergence time are illustrated in Figure 15, where the convergence time to execution of ADR and LoRaBB in each scenario evaluated is illustrated. We observed a minimal difference in convergence time, primarily influenced by the LoRaBB processing time, as we can observe in the convergence time for approximately 20 packets transmitted by ADR and 8 packets for LoRaBB. Therefore, the total convergence time of the proposed algorithm was slightly more than double compared to the ADR result due to the number of packets transmitted by LoRaBB, which was slightly more than 40 packets due to the iterations of the binary search.

On the other hand, in the qualitative analysis, the LoRaBB algorithm consistently chose parameters SF = 11, BW = 250, and TP = 14 in both experiments, contrasting with ADR, which assigned SF = 10, BW = 125, and TP = 20. We highlight that in both experiments ADR did not decrease the TP value. Due to foliage across the forest, the SNR value, used in ADR, is usually poorer in these environments, which leads the ADR to keep higher TP values.

LoRaBB held an advantage by selecting specific parameters which resulted in a ToA, compared to the ADR result, 206.84 ms and 247.80 ms, respectively (results extracted with LoRa calc: https://www.semtech.com/design-support/lora-calculator, accessed on 1 July 2024). As stated by Križanović et al. [39], ToA is fully related to device energy consumption, where higher ToA implies more energy consumption by the transmitter device.

The performance evaluation shows LoRaBB can perform similarly to ADR in terms of SF parameter selection. Since higher SF can handle more information on its chirp and consume more device energy, it also can lead to fewer messages sent by day (considering the fair use policies and regulated duty cycle limits) [39]. Although communication operations involving SF are quite exponential, we believe this does not significantly impact operations related to remote monitoring systems, such as sensor-based monitoring of water quality—the main use case of this research—since there is no requirement for devices to send a large volume of messages daily in such environments.

In addition, as expected, the LoRaBB reached a lower TP when compared to the ADR result. While ADR is not properly indicated for TP parameter selection due to the algorithm and scenario properties, LoRaBB can handle TP selection, choosing TP =14 in both experiments evaluated. It is worth mentioning that, as stated in [40], the energy consumption can increase more than 340% when TP goes from 13 to 20 dB considering the Semtech chip datasheet and 38% when TP goes from 17 to 20. The differences between the literature and the chip datasheet support our findings that LoRaBB can lead to a minimum device energy consumption.

**Figure 16:**
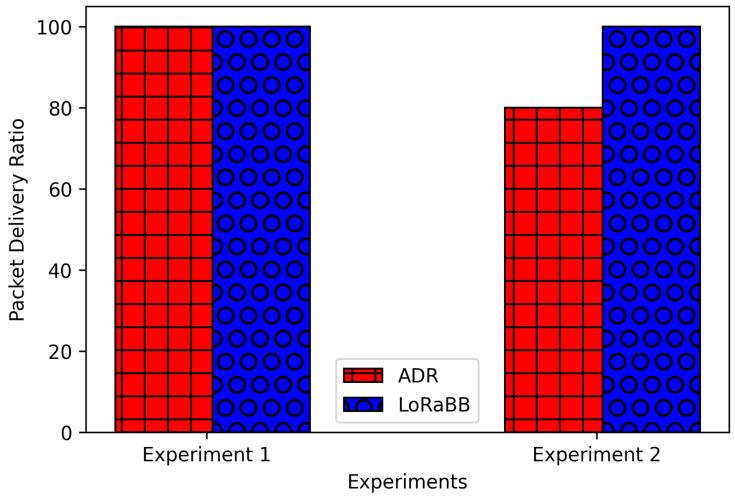
Packet delivery ratio.

Further, analyzing packet delivery performance, as illustrated in Figure 16, the ADR had the worst performance on experiment 2, reaching 100% and 80% for each experiment scenario, while LoRaBB reached a 100% PDR in such cases. Even in a scenario of a relevant short distance for LoRa communication, ADR did not reach a 100% PDR.

One of our hypotheses is that, as ADR uses the SNR as the basis of its algorithm, the SNR was verified to be a metric of minor applicability in forest scenarios, which is corroborated by the investigation by Ansah et al. [36]. where the authors found the received signal power deviated due to scattering caused by tree trunks and the canopy. Moreover, our results follow the theory that higher values of SF had longer ToA and lower minimum receiver sensitivity, as LoRaBB achieved SF =11, while ADR =10, which could result in the high variance in the received signal power found in [36], impacting the PDR.

Although minimizing the TP value selected for cost-effective deployment in non-critical and non-real-time applications, LoRaBB’s chosen parameters are preferred due to device energy constraints.

## 6. Conclusions and Future Work

This work introduced a novel algorithm for LoRa parameter selection based on the binary search methodology entitled LoRaBB. LoRaBB aims to adapt the LoRa parameter selection to the deployment scenario based on the previous scenario characterization. Further, the conditions of the deployment considered an end node-to-gateway communication in a star network topology.

Using a pre-built *R*-array, LoRaBB can identify the relationship between key parameters (SF, BW, and TP) used in LoRa communication and the major metrics evaluated by LoRa for transmission quality: RSSI, SNR, and packet delivery ratio (PDR) for each deployment scenario. Further, this characterization was applied and evaluated in forest scenarios to support environmental monitoring systems used in the context supported by this project.

We therefore conclude that our approach can reduce other device-related expenses due to the self-healing and self-configuration settings. LoRaBB chose more energy-efficient parameters than the traditional ADR algorithm, resulting in a lower Time on Air and a lower TP, despite taking a longer time to find the suitable parameter combination. It also selected more feasible parameters for LoRa communication in forest environments, such as the Amazon rainforest, extending the device’s lifespan in monitoring scenarios in forest environments.

On the other hand, the long-range capabilities and gateway configurations for managing multiple devices with different LoRa parameters emerge as a new issue in this context. As a next methodological step, we intend to analyze other environmental factors and climate change and how LoRaBB and other LoRa parameter selection algorithms can handle these situations dynamically. Additionally, to show how gateways handle the parameter changing, we consider testing over different window parameter selections or based on recent packet losses.

In future work, we might adapt LoRaBB considering different climate conditions, such as the rainy periods in the Amazon rainforest. Since this work was evaluated in the summer, the rainy period did not impact the characterization and results. Finally, we highlight that the LoRaBB must be compared to novel machine learning approaches for LoRa parameter selection. Since it is related to other parameters, such as energy spent by the method and algorithm complexity, this study did not address these techniques.

## Figures and Tables

**Table 1:** Summary of research studies on LoRa parameter selection.

Authors	LoRa Parameters Used	Scenario	Missing Points
Gao et al. [24]	SF, CR, BW	Simulated	The authors presented a mathematical model to achieve max–min fairness of energy efficiency based on LoRa parameter selection considering free-space path loss. Nevertheless, the authors did not consider any impact of the environment on signal attenuation in the mathematical modeling.
Angrisani et al. [25]	SF, CR, BW	Real Scenario	The authors verified the robustness of the LoRa communication protocol versus different levels of white Gaussian noise based on SF, CR, and BW values. Despite demonstrating the robustness of LoRa in different noise scenarios, the impact of environment attenuation factors were not fully evaluated.
The Things Network (TTN) [26]	SF	Simulated	In the ADR TTN white paper, the ADR is based on the link budget capacity to update SF. In addition, many aspects of algorithm design and parameter values were not fully addressed, and even the details of the mathematical modeling were not.
Serati et al. [27]	SF, TP, CF, CR	Simulated	The authors applied simulations using the Oulu Lora Path Loss model based on real urban scenarios. Their modified ADR version considered mobile and static end devices. Thus, the path loss model used in their experiments may not be suitable for performance evaluation considering communication within vegetation and forests.
de Jesus et al. [28]	SF, SNR	Simulated	The authors evaluated their experiments by comparing SF and SNR performance using free-space path loss, which may not be suitable for reflecting behavior in forest communications.
Khalifeh, Shefaa, and Darabkh [29]	SF	Simulated	The authors presented an algorithm to select SF parameters based on rate demand. In their simulations, they applied a white Gaussian noise in path loss to simulate lossy urban environments. Other attenuation factors were not addressed in the algorithm design.
ML techniques [13,32]	SF, CR, BW	Simulated	The machine learning-based algorithms proposed by [13,32] lack a detailed specification of the path loss model used in their simulations. While these studies considered multi-device communication channel interference, they did not comprehensively evaluate environmental attenuation factors.

**Table 2:** Default parameters (DPs) used in LoRa transmission in first node–gateway interaction.

Parameter	Value
Spreading Factor	12
Bandwidth	125 kHz
Transmission Power	20 dBm
Code Rate	4/5

**Table 3:** Characterization experiment settings.

Parameter	Values
Spreading Factor	$\left[7,8,9,10,11,12\right]$
Bandwidth	$\left[125.00,250.00,500.00\right]$ kHz
Transmission Power	$\left[10,12,14,16,18,20\right]$ dBm
Coding Rate	4/5

**Table 4:** Detailed example of LoRaBB binary search phase. Each step shows the parameter combination in each test with the calculated *R* value, the expected *R*-array value for the current parameter combination, and the binary search step.

Step	Combination	SF	BW	PT	Calculated *R*	$R[C_{k}]$	Move
1	$C_{5}$	8	500	20	0.30	0.50	Move Right
2	$C_{8}$	12	125	20	0.95	0.80	Move Left
3	$C_{6}$	10	500	20	0.75	0.70	Accept

## Data Availability

Data are contained within the article.

## Abbreviations

The following abbreviations are used in this manuscript:

LoRa	Long Range
TP	Transmission power
BW	Bandwidth
SF	Spreading factor
CR	Coding rate
ADR	Adaptive Data Rate
ToA	Time on Air
RSSI	Received signal strength indicator
SNR	Signal-to-noise ratio
PDR	Packet delivery ratio
